# The virtual indigenous data science academy: development of a summer program in data science for tribal college students

**DOI:** 10.3389/fpubh.2026.1709106

**Published:** 2026-02-12

**Authors:** Corey B. Smith, Melanie Nadeau, Collette Adamsen, S. Cristina Oancea, Brent Voels, Adrienne Salentiny, Lynn Mad Plume, Michael Herbert, Mandy Guinn, Lyle G. Best, Emily Biggane

**Affiliations:** 1 Department of Pathology, School of Medicine and Health Sciences, University of North Dakota, Grand Forks, ND, United States; 2 Department of Population Health, School of Medicine and Health Sciences, University of North Dakota, Grand Forks, ND, United States; 3 Department of Indigenous Health, School of Medicine and Health Sciences, University of North Dakota, Grand Forks, ND, United States; 4 Department of Population Health, Center for Rural Health, School of Medicine and Health Sciences, University of North Dakota, Grand Forks, ND, United States; 5 Department of Science, Technology, Engineering and Math, Turtle Mountain Community College, Belcourt, ND, United States; 6 School of Medicine and Health Sciences, University of North Dakota, Grand Forks, ND, United States; 7 Center for Indigenous Health-Great Plains Hub, Johns Hopkins Bloomberg School of Public Health, Rapid City, SD, United States; 8 University Analytics and Planning, University of North Dakota, Grand Forks, ND, United States; 9 Department of Environmental Science, United Tribes Technical College, Bismarck, ND, United States; 10 Genetics and Pre-eclampsia Study, Natural Science Department, Turtle Mountain Community College, Belcourt, ND, United States; 11 Intertribal Research and Resource Center, United Tribes Technical College, Bismarck, ND, United States

**Keywords:** data science, indigenous, online, tribal college, workforce

## Abstract

Data science skills are essential for advancing knowledge of health inequities in marginalized and medically underserved populations. As of 2021 there were no programs in data science in the Tribal Colleges and Universities in North Dakota and the neighboring states. The Virtual Indigenous Data Science Academy was established in 2021 by the University of North Dakota School of Medicine and Health Sciences in collaboration with three North Dakota Tribal Colleges to introduce Indigenous students to the core elements of data science. The Virtual Indigenous Data Science Academy teaching faculty designed, developed, delivered, and evaluated a culturally-informed online synchronous summer program consisting of ten learning modules. The Virtual Indigenous Data Science Academy was delivered synchronously over three consecutive summers (2022–2024) to a total of 48 Tribal college students with diverse backgrounds and academic interests. More than 70% of student participants met the skills-based learning objectives of the Academy. Results of the summer program student evaluations demonstrated higher than average ratings of satisfaction with the instruction. The VIDS Academy represents a promising model for the delivery of instruction in data science and Science, Technology, Engineering, and Mathematics-related fields that address the local workforce and healthcare needs of Tribal communities.

## 1 Introduction

Data science skills are essential for advancing knowledge of factors that reinforce health inequities in marginalized and medically underserved populations. A major contributor to health inequities is the lack of a diverse biomedical research workforce proficient in data science. Programs that introduce students to computational approaches, role models, resources, and career opportunities in data science are needed to address the under-representation of racial and ethnic minorities in this workforce (1). Interaction with Indigenous researchers with lived and Tribal research experience is crucial for building student capacity to identify meaningful research gaps in Tribal communities (2).

Despite the critical need, few minority-serving institutions offer specialized data science training (3). The Tribal Colleges and Universities (TCUs) in North Dakota (ND) and the surrounding region have no existing data science programs. While TCUs have a long history of preparing students for Science, Technology, Engineering, and Mathematics (STEM) fields that address local workforce needs, TCUs are principally teaching institutions not designed to support research development. TCUs across the US provide culturally-relevant, high-quality educational opportunities for Native students and others to earn degrees at relatively low cost. In rural ND, TCUs also serve as vital links to social and economic opportunities. None of the five TCUs in North Dakota offer courses or specialized training in health data science in spite of the need for this expertise by Tribal Health Organizations (THOs) (4). National efforts, including the 2021 National Academies Town Hall on Advancing STEM Workforce Preparation and Research Capacity at Tribal Colleges and Universities, have emphasized the urgent need for culturally responsive strategies to strengthen STEM education and research infrastructure in TCUs. The VIDS Academy responds to this call by providing a virtual, culturally grounded data science program designed in partnership with Tribal Colleges to build technical skills while affirming Indigenous knowledge systems.

Building on these national recommendations and addressing the identified gap, the Virtual Indigenous Data Science (VIDS) Academy was established to address this gap in data science education for Indigenous students in North Dakota. With funding from the NIH-supported North Dakota IDeA Networks of Biomedical Research Excellence (INBRE), the Department of Indigenous Health at the University of North Dakota (UND) School of Medicine and Health Sciences (SMHS) and three ND Tribal Colleges collaborated to develop a week-long summer training program introducing ND Indigenous students to the core principles of data science, including data management, analytic skills, and tools for health research. The current study reports on program development from inception in 2021 through 2024.

The VIDS Academy was intentionally designed as a synchronous virtual program to overcome geographic and resource barriers faced by Tribal College students. By combining online delivery with experiential learning activities—such as hands-on data analysis, interactive breakout sessions, and culturally grounded discussions—the program provided an accessible and engaging approach to STEM and public health education. This design aligns with emerging evidence that virtual and experiential learning models are effective for underrepresented students in STEM and public health fields, particularly when paired with culturally sustaining pedagogy and community-relevant content (5).

The VIDS Academy aligns with several established frameworks that support Indigenous student learning and guide culturally grounded educational design. The program reflects key principles of Culturally Sustaining Pedagogy, which emphasizes sustaining and expanding students’ cultural identities within academic spaces (6). By integrating community-relevant examples, Tribal health priorities, and instruction from Indigenous faculty and peer mentors, the VIDS Academy affirms Indigenous knowledge systems while strengthening data-science proficiencies.

The program was also informed by ideas central to Decolonizing Methodologies, which call for centering Indigenous ways of knowing, challenging extractive research practices, and ensuring that educational activities serve community interests (7). The participatory planning approach—particularly the involvement of Tribal College faculty and the Curriculum Advisory Committee—reflected these decolonizing commitments and ensured that course design was collaborative, culturally grounded, and aligned with Tribal priorities (8).

Finally, the structure and evaluation of the VIDS Academy align with Indigenous Evaluation frameworks, which emphasize relationality, accountability, cultural relevance, and collective meaning-making (9). The use of reflective writing, talking circles, and ongoing iterative input from Tribal College partners reflects these values and provided mechanisms for culturally grounded assessment and continuous program refinement (10).

In alignment with these guiding frameworks, the VIDS Academy also incorporated the CARE and FAIR Principles to model ethical and culturally grounded approaches to data use. While the FAIR Principles provided structure for teaching the technical foundations of locating, organizing, and documenting datasets in ways that promote findability and reusability, the CARE Principles emphasized Indigenous rights to collective benefit, authority to control data, responsibility, and ethical use within Tribal contexts (11). Integrating these principles ensured that students learned not only how to work with data technically, but also how data practices intersect with sovereignty, relational accountability, and community well-being—echoing core ideas of Indigenous Evaluation and decolonizing approaches to research (7, 9). Together, the CARE and FAIR frameworks provided a balanced foundation that supported both proficient data management and culturally respectful, sovereignty-affirming data literacy. This initiative aimed to support ND Indigenous student participation in data science by developing a virtual training course that reflects TCU students’ background and culture. A participatory design approach was adopted for the development of the curriculum by the UND SMHS leadership team, with input from an external Curriculum Advisory Committee (CAC) (8). It was essential that TCU students benefit from the synergies created using a flexible process involving the culturally-grounded STEM programming of TCUs and UND’s computer science and health sciences education and research training capabilities.

## 2 Overview of pedagogy

### 2.1 Curriculum model

The curriculum was developed using the Dick and Carey model of systematic instructional design, which emphasizes the identification of outcomes before aligning learning objectives, assessments, and instructional strategies (12). Outcomes were specified using the Needs Assessment and Interest Survey (described below) and the reflective expertise of teaching faculty in the program and associated Tribal colleges. The leadership team and CAC workshopped these to create sixteen performance objectives. These objectives, along with the barriers and resource needs identified in the survey, informed the development of assessments and instructional strategies using resources students were familiar with in a challenging but feasible schedule and format.

### 2.2 Needs assessment and interest survey

Prior to program development, a Needs Assessment and Interest Survey was emailed to twelve Tribal college faculty from the five ND Tribal Colleges who attended informational meetings about the program. The nineteen-question survey asked about faculty background, Tribal college learning environment, students’ access to computing resources, and faculty opinions on topics of potential student interest in data science. Survey results informed curriculum design and program implementation.

## 3 Faculty, learning environment and pedagogical format

### 3.1 Leadership team

The VIDS Academy leadership team consisted of six core UND SMHS teaching faculty, one instructional designer, one evaluator, and a total of five peer mentors over three summers. The six core teaching faculty hold advanced academic degrees and training in biostatistics, epidemiology, molecular biology, and biomedical informatics with a combined 105 years of research and/or teaching experience. Four of the six faculty have decades of experience working directly with Tribal communities. All team members contributed to the development of the curriculum with advisement from the instructional designer. Indigenous peer mentors were recruited from UND and one TCU to provide academic support and technical assistance to students and faculty. All peer mentors were senior undergraduate students with majors in mathematics or science disciplines. Administrative staff from the UND SMHS Department of Pathology provided additional programmatic support. Each year, an independent evaluator was contracted to evaluate the program.

### 3.2 Curriculum advisory committee

A Curriculum Advisory Committee (CAC) was formed early in Year One (2022) of the VIDS Academy. Prospective members were recruited from STEM faculty at each of the five ND Tribal Colleges. Seven faculty from three of the five participating Tribal Colleges accepted the invitation to serve on the Committee. Committee members advised on the structure, content, and delivery of the curriculum and ensured that it was culturally and academically responsive to the needs of student participants.

### 3.3 Timeline

The VIDS Program launched in November 2021. The UND SMHS leadership team met monthly from November through the summer months each program year to plan, discuss curriculum development and implementation considerations, and address potential programmatic challenges. The CAC began meeting in February of Year One (2022) to review needs assessment findings, recommend strategies for student recruitment and support, and guide the development and evaluation of the curriculum. The CAC also participated in the planning and course revision in Years Two and Three.

### 3.4 Curricular structure

The course curriculum consisted of ten instructional modules guided by sixteen learning objectives. Each module included topics, learning objectives, recorded lectures (if applicable), readings, activities, and additional resources curated and developed by core teaching faculty in response to the Needs Assessment and Interest Survey and CAC guidance. A team-teaching framework was used for most modules; two introductory modules were taught independently. All modules were evaluated and revised with teaching faculty expertise and CAC oversight; recurring meetings provided continuous feedback. A formal evaluation was completed at the end of each program year.

The primary aim in Year One (2022) was to develop a curriculum that leveraged the existing UND SMHS expertise, including Indigenous health research scholars. In Year Two, the curriculum was revised to better represent ND TCU faculty partners’ research interests and expertise. Most TCU faculty involved with the VIDS Program directed programs focused on topics in the environmental sciences. This resulted in a thematic shift away from the Year One focus on cancer and -omics research to examining climate change effects on mosquito-borne infections and public health.

### 3.5 Student recruitment

A multi-pronged approach was adopted for student recruitment. Any Tribal college student in good academic standing was eligible to enroll in the program. Faculty advisors identified by students at registration were contacted to determine eligibility. Registration was limited to thirty Tribal college students annually for 2022–2024. In Year One, the program developed a website with course description and downloadable brochure for recruitment and registration of interested students. The program was also promoted through (i) brochures with QR codes distributed at an annual regional Tribal college research symposium each spring, (ii) Tribal College Facebook pages, and (iii) CAC faculty referring students to the program. In Year Three (2024), the geographical reach of the program extended beyond ND to a national audience through student recruitment at the annual American Indian Higher Education Consortium conference. A VIDS Academy Scholar Agreement was instituted requiring students to participate in all course learning activities. Each student who completed the course received $1,000 to offset the possible loss of income from summer employment due to program participation.

### 3.6 Instructional tools and methods

All students were required to have access to a computer installed with webcam, Microsoft Excel software, and reliable internet connection. The VIDS Academy was delivered synchronously over the Zoom videoconferencing platform. During Years One and Two, students could also use tablets and mobile phones in place of computers. In Year Three, it became necessary for students to actively engage with the course material using multiple software applications simultaneously. Consequently, mobile devices were not approved for course participation. Google Docs was used for daily attendance sign-in, reading the daily schedule, and retrieving course-related hyperlinks. Lectures, readings, and additional course resources were accessed from the UND Blackboard learning management system. In Year Three, one of the Tribal Colleges served as a host site where students joined from campus. The expectation was that assembling students at their academic institutional home might increase engagement.

### 3.7 Delivery of curriculum

In Year One, the 1,770 min curriculum was delivered over four and a half days. In Year Two, the faculty determined that more time was necessary for adequate coverage of topics. Therefore, the course was extended an additional 150 min in Years Two and Three. Students met on the morning of Day One for faculty and student introductions, establish ground rules, participate in a pre-course survey, and review the course curriculum. A weekend break separated Days One and Two. Days Two through Four covered the remaining modules Two through Ten. Students met for 7 h each day, which including a one-hour lunch break and occasional short breaks.

Instructional time for modules in Year Two amounted to 735 min (see Table 1) and 915 min in Year Three (see Table 2). An additional 180 min of instructional time, not reflected in the modules, was incorporated to address student questions. Time was also reserved for students to review a scientific paper on data mining techniques to predict West Nile Virus (13).

**Table 1:** Vids 2.0 academy curriculum, June 2023.

Day	Module	Length (in minutes)
Day 1	1: Foundations of data science	30
Day 2	2: Cultural aspects of data collection and use	120
	3: Introduction to mosquitoes, ecology, and population dynamics	45
	4: Overview of data sources	90
Day 3	5: Programming and tools for data science	30
	6: Understanding distributions, statistics, and hypothesis testing	30
	7: Data management and processing	120
Day 4	8: Data exploration and visualization	50
	9: Analytics and big data in healthcare	90
	10: Introduction to machine learning	130
Total time		735

**Table 2:** VIDS 3.0 academy curriculum, July 2024.

Day	Module	Length (in minutes)
Day 1	1: Foundations of data science	30
Day 2	2: Cultural aspects of data collection and use	120
	3: Introduction to mosquitoes, ecology, and population dynamics	45
	4: Overview of data sources	150
Day 3	5: Understanding distributions, statistics, and hypothesis testing	120
	6: Data management and processing	100
	7: Programming and tools for data science	70
Day 4	8: Analytics and big data in healthcare	90
	9: Introduction to machine learning	130
	10: Communicating with data	60
Total time		915

Six of the modules in Year Two were reordered in Year Three to promote greater continuity and integration of the curriculum. Time allocated for Module Four in Year Two nearly doubled in Year Three to accommodate additional content. More time was reserved for modules on Programming Tools for Data Science and Understanding Distributions, Statistics, and Hypothesis Testing. The amount of time for Module Seven in Year Two was reduced in Year Three. Module Eight on data visualization in Year Two was revised, renamed, and moved to the end of the week in Year Three.

At the end of each day, the faculty met for a fifteen-minute debrief to discuss any needed mid-course corrections. On Day Five of each year, the Academy concluded with a half-day session that featured a Career Spotlight presentation by an Indigenous research scholar, discussion of future educational opportunities, and course evaluation.

## 4 Results

### 4.1 Needs assessment and interest survey

Eleven faculty from four of five Tribal Colleges in ND completed the Needs Assessment and Interest Survey in Year One (2022). Respondents reflected a range of expertise in a variety of STEM fields, which included the natural sciences, information technology, and others. Academic rank of the faculty also varied. Six faculty had a decade or more of Tribal College service. Faculty reported that synchronous online delivery of the course would be most effective for students. The faculty noted that family and job commitments were the most likely conflicts to interfere with course participation by the students. All faculty agreed that payment incentives would be the most effective incentive for course completion. Faculty reported that most Tribal College students had access to computer and/or computing resources at their institution. Ten faculty indicated that students were familiar with the use of Microsoft Excel for working with data. Two faculty reported that Python had been used by students at their Tribal college. No faculty member reported that their students were familiar with R, SAS, or SPSS. Sports, entertainment and environmental science were ranked as the most engaging domains of interest for teaching data science.

### 4.2 Student participants

Seventy-five students registered for the Academy over 3 years. Thirty students registered for the Year One (2022) pilot. Twenty-five students registered in Year Two and twenty students registered in Year Three. Of those who registered, 48 (64%) students participated in one or more course modules (see Table 3); 36% cancelled their registration. Reasons cited for course cancellation included work schedule conflicts or illness. No significant differences in age, gender or home Tribal College were found between students who registered and those who participated.

**Table 3:** Characteristics of VIDS academy participants, 2022–2024, (*N* = 48).

Characteristic	VIDS 1.0 (*N* = 19)		VIDS 2.0 (*N* = 16)		VIDS 3.0 (*N* = 13)		Total (*N* = 48)	
	*n*	%	*n*	%	*n*	%	*n*	%
Age (years)								
15–25	4	21.0	6	37.5	5	38.5	15	31.3
26–34	11	58.0	8	50.0	6	46.1	25	52.1
35+	4	21.0	2	12.5	2	15.4	8	16.6
Gender								
Woman	13	68.4	11	69.0	9	69.2	33	68.7
Man	6	31.6	5	31.2	2	15.4	13	27.1
Two spirit	0	0.0	0	0.0	2	15.4	2	4.2
Non-binary	0	0.0	0	0.0	0	0.0	0	0.0
Gender queer	0	0.0	0	0.0	0	0.0	0	0.0
Another gender identity	0	0.0	0	0.0	0	0.0	0	0.0
Prefer not to answer	0	0.0	0	0.0	0	0.0	0	0.0
Race^a^								
American Indian/Alaska Native	17	89.5	16	100.0	12	92.3	45	93.8
Black or African American	1	5.3	0	0.0	1	7.7	2	4.2
White	1	5.3	1	6.3	1	7.7	3	6.3
Native or Pacific Islander	0	0.0	0	0.0	0	0.0	0	0.0
Asian	1	5.3	1	6.3	0	0.0	2	4.2
Ethnicity								
Hispanic/Latin (o/a/x)	0	0.0	0	0.0	0	0.0	0	0.0
Not Hispanic/Latin (o/a/x)	19	100.0	16	100.0	13	100.0	48	100.0
Disability status								
Yes	0	0.0	1	6.3	1	7.7	2	4.2
No	16	84.2	11	68.7	10	77.0	37	77.0
Prefer not to answer	3	15.8	4	25.0	2	15.4	9	18.8
Tribal college								
Tribal college 1	5	26.3	9	56.3	5	38.5	19	39.6
Tribal college 2	1	5.3	0	0.0	0	0.0	1	2.1
Tribal college 3	2	10.6	0	0.0	0	0.0	2	4.2
Tribal college 4	7^b^	36.8	6	37.5	3	23.1	16	33.3
Tribal college 5	4	21.0	1	6.2	1	7.7	6	12.5
Other	0	0.0	0	0.0	4	30.7	4	8.3

a Students of mixed race could select more than one race. Column totals may exceed the number of student participants in a given cohort.

b One student participant was a Tribal college faculty member.

Students were predominantly women (*n* = 33, 68.7%) and young adults between 26 and 34 years of age (*n* = 25, 52.1%). Across all 3 cohorts (2022–2024), nearly all students self-identified as American Indians/Alaska Native (*n* = 45, 93.8%), including three (6.3%) of mixed race. Individuals could self-identify as more than one race at registration. With few exceptions (*n* = 6), Indigenous students were affiliated with northern plains Tribes. Two of the five ND Tribal Colleges accounted for nearly three-quarters of all students (*n* = 35). Three students from out-of-state Tribal colleges participated in Year Three. One advanced high school student from a Tribal community also participated.

VIDS Academy students represented diverse academic fields (see Supplementary material for major areas of study of VIDS participants).

### 4.3 Program evaluation

The purpose of the VIDS evaluation was to assess the effectiveness and impact of the program on students. The evaluation of the program entailed both formative and summative components to ascertain whether the objectives of the program were achieved. Evaluation methods varied from year to year as the learning objectives also evolved with the growth and expansion of the program. The core values of Indigenous evaluation were observed throughout all evaluation activities (9).

#### 4.3.1 Year one

The Year One pilot aims were to develop and launch the course curriculum. Student evaluation activities consisted of: (a) pre- and post-assessments; (b) one-minute reflective writing assignments to assess participant experience of each module; and, (c) a talking circle as summative assessment for students to reflect on their experience in the program. The pre-post assessments, each of which consisted of five questions, assessed whether the course met student expectations, appraised changes in views on the importance of data for Native communities, and evaluated familiarity with Indigenous research experiences. The writing assignment asked students to identify what they liked or disliked about each module and to suggest improvements. Key takeaways identified by the students in the reflective writing assignments informed future course delivery and drove continuous curriculum improvement. The talking circle was a facilitated discussion in which students shared experience in a culturally responsive way. Talking circles are an Indigenous method allowing all voices to be heard (10). Details of the discussion were captured as meeting notes by one of two evaluators. The talking circle was not audio or visually recorded. Evaluators conducted content analysis of the talking circle notes to identify key themes based on Indigenous Data Sovereignty principles and practices (11).

##### 4.3.1.1 Pre-post assessment

Initially, students stated in a variety of ways that they expected to learn methods for gathering and interpreting data. Many expressed a particular interest in how to use data for the benefit of Indigenous people. At course conclusion, students reported the program was informative but noted that limited Indigenous-specific data sometimes hindered engagement with course material. Students reported little to no experience interacting with health data or with the field of data science, generally. However, a few students reported some experience using software packages, such as Excel, R, and ArcGIS. At the conclusion of the course, students reported better understanding of the importance of health-related data for Native communities. When asked to speculate why understanding data and its application are important, students noted that data are important for Indigenous people and Tribal nations because it “promotes data governance.” Two additional questions assessed familiarity with negative and positive research conducted with Native people. Initially, three-quarters of registrants were unfamiliar with any negative research experiences involving Native people, while 35% (*n* = 7) reported that they were not familiar with any positive experiences either. Course participation shifted student awareness of research experiences with Native people. Awareness of negative (*n* = 13; 67%) and positive research experiences (*n* = 8; 44%) increased at program completion.

##### 4.3.1.2 Reflective writing assignments

Overall, students found modular content beneficial for understanding data science. The probability and statistics module brought into focus both the major challenges and benefits of data science. Students reported being overwhelmed at first by the amount of material, but also acknowledged that this module increased their comfort level with the quantitative aspects of the course in ways that positively affected how they viewed the other modules. For instance, students pointed to how the use of real-world examples of data drawn from Native populations helped them to better appreciate the relevance of math concepts. While students appreciated faculty presentations highlighting Indigenous applications of data science, they requested more examples of Indigenous-specific data and research. Incorporating more hands-on activities was an additional finding across all modules.

##### 4.3.1.3 Talking circle

Four themes emerged from the Talking Circle: (i) Timing and length of course; (ii) cultural relevance; (iii) empowerment and representation; (iv) community and culture. While students valued course content, they recommended extending it over 2 weeks and splitting longer modules into multiple sections. Students maintained that the modular content on math concepts would have been easier to retain, and result in greater engagement with the material, if split into two sections. Participants expressed appreciation for the cultural relevance of the course. For example, students mentioned the use of technology for mapping traditional dancing as well as the research presentations using Indigenous data. Students desired even greater integration of culturally-grounded content into the curriculum. An extension of this theme was observed in the ways students talked about feeling “hopeful” and “empowered” by listening and seeing Indigenous professionals talk about their research. Finally, student participants stressed that community and culture were the most significant motivators for learning data science, referencing their families and communities as examples.

#### 4.3.2 Year Two

The Year Two evaluation was partially completed due to the unexpected departure of the program evaluator from the project. Consequently, program evaluation for Year Two is limited to data from the reflective writing assignments and the talking circle.

##### 4.3.2.1 Reflective writing assignments

Several themes from the writing assignments in Year One were echoed in Year Two. For instance, students appreciated the module on understanding statistics and hypothesis testing. One student, who expressed the sentiment of other cohort members, “I liked how every topic was explained and with an example.” Integrating mosquito population dynamics with statistics was especially well-received. Other modules that students found particularly beneficial included coverage of the selection and use of software as well as how to examine, evaluate, and prepare data for analysis. Modules using extensive visual data representations were “informative,” especially for unfamiliar content. One student remarked that the graphs and charts used in the module which highlighted the process of collecting data from Tribal elders helped her to “…understand what’s happening to tribal elders.” As in Year One, students underscored their preference for more active aspects of the course. Two students suggested one module could be improved by incorporating an activity. Another expressed more positively: “Working in breakout groups and answering questions was my favorite part.” However, even for interactive portions, some students wanted more preparation time for activities. Differences in Excel experience, which proved challenging for some students, became apparent early in the course when working with mosquito data. While most students were able to complete the activity without assistance, a few students mentioned the lesson pacing was “too quick” and that they “lost track” of where they were in the assignment.

##### 4.3.2.2 Talking circle

Themes in Year Two partially overlapped with Year One. Course length and cultural relevance again emerged as major themes. Most students recommended that more time be allocated to each of the modules. This was consistent with the feedback that was received from the reflective writing assignments. Students again suggested extending the overall length of the course from 1 week to 2 weeks “to cover the topics better.” And again in Year Two, students appreciated the incorporation of “lectures of Indigenous guest speakers” and opportunities to “learn more about Indigenous data science.” Unlike Year One, students expressed course management concerns in Year Two. Three students cited virtual delivery of the course as a barrier to participation. One student expressed it this way: the course “…would be better in a classroom where the teacher can show you.” Two students mentioned the initial technical challenges accessing the learning management system. Several students preferred greater use of breakout rooms “to help one another” with homework. Despite these concerns, students agreed the course was a “great introduction” to data science and would “recommend to others.”

#### 4.3.3 Year Three

The Year Three evaluation consisted of an assessment of student learning and a survey of student feedback at the end of the program.

##### 4.3.3.1 Student learning

Evaluation of student learning consisted of formative and summative assessments. The formative assessment included 4 multiple choice, true/false, or short-answer questions administered after module (except Module 2). Correct answers to questions were reviewed in real-time with students. Summative assessment questions determined the extent to which students mastered key concepts. Additional methods and the results of the VIDS 3.0 summative learning assessment are available as Supplementary material.

Upon course completion, students reported perceived gains in skills. With one exception—distinguishing between data formats—students reported improved skills from Academy participation. The greatest gains occurred in the application of the data science life cycle, demonstrated understanding of the public health importance of mosquito-borne disease, and, understanding the use and limitations of algorithms with real-world data. Slightly smaller gains were observed involving activities that required use of software for data exploration (see Table 4).

**Table 4:** VIDS 3.0 self-reported improvements in data science skills (*N* = 13).

Skill	M	SD	Gain (%)
Application of the data science life cycle	3.2	0.60	92.3
Public health perspective of mosquito-borne disease	3.6	0.65	92.3
Locating, downloading, and evaluating public data sources	3.3	0.95	84.6
Telling the difference between data formats	2.9	0.76	69.2
Steps using software to explore a data file	3.2	0.99	77.0
Choosing a programming language or appropriate software for statistical analysis	3.2	0.73	84.6
Matching a chart or line graph to data type	3.5	0.97	84.6
Use and limitations of algorithms with real-world data	3.6	0.65	92.3
Understanding how data can be communicated in an impactful way for Indigenous communities	3.5	0.78	84.6

Responses based on 4-point Likert scale of (1) Not at all, (2) Somewhat, (3) Moderate amount, and, (4) A lot.

##### 4.3.3.2 Student feedback

Students provided feedback on program content and instructional effectiveness after each module and at the end of the course. The results of the post-assessment are shown in Table 5.

**Table 5:** Vids 3.0 themes identified in student post-assessment (*N* = 13).

Question	Theme	Comments
1. What was your favorite part of the VIDS Academy?	Skill development	*I liked the interaction between students and faculty…* *I liked the hands-on activities…* *…lots of help and assistance with projects* *…finding data about mosquitoes*
	Indigenous speakers	*The presentations on Native-based research* *The Indigenous aspect* *The guest speakers and lecturers…on Indigenous communities*
2. What was your least favorite part of the VIDS Academy?	Cadence	*…sitting for a long time…* *…sitting for 7 h a day* *Need more breaks…* *Having to sit there for a long period of time and listen.*
3. What else would you like to learn about data science?	Applications	*Types of data I did not understand…* *How to get into more…graphing and downloading the data* *…learn more about the data collection process and more about how to approach setting up research methodology* *…how statistics are used in everyday life*
4. What recommendations do 5. You have for future versions of the VIDS Academy?	Duration	*I would like it to be extended…* *I recommend it being stretched into a 10 day course* *…shortening the days and doubling the range of days…* *Make the days shorter but the weeks longer…*
	Active engagement	*More interactive parts like making graphs/collecting data…* *More activities and games…*

Student post-assessment comments aligned with reflective writing themes across all 3 years. Presentations from Indigenous speakers were among the most well-liked parts of the Academy. Activities involving application of concepts were also favored by the students. Conversely, the least favorite aspects of the program were the uninterrupted periods of didactic instruction. Students recommended extending the future courses to allow for more interaction.

## 5 Discussion

This report summarizes a three-year experience of a ND university-Tribal college partnership aimed at developing a summer course in data science for ND Tribal college students. One of the early team decisions focused on the amount of time allocated for delivery of the course. Although two or more weeks for delivery of the course had been considered, the UND leadership team and Tribal college faculty agreed that a shorter online course would improve retention. A longer course might have discouraged enrollment or caused absences, as many Indigenous students engage in cultural or field activities during the summer months. In Year One, recruitment exceeded expectations. In fact, two students had to be waitlisted. In Year Three, the window for recruiting students was moved forward as many students make commitments for summer employment or enroll in other STEM programs earlier in the year.

The core teaching faculty developed the curriculum, which was organized as a series of modules with each module covering a set of related topics. Longer modules—four, six, and nine—had to be split into smaller units of instruction The modular organization of the curriculum functioned reasonably well. Learning objectives and course content for each module were reviewed annually. Revisions to the content, sequence, and delivery of the modules were made each year, based on student feedback. While individual module objectives were slightly modified in Years Two and Three, the overall course objectives remained consistent.

This is the first known course of its kind delivered in a distance learning format. This necessitated the development of course policies governing the use of technology as well as the overall learning experience. Ground rules were established that democratized the learning environment so that students could safely ask questions and engage with the material. Early on, the pacing of the course needed to be adjusted to accommodate individual learning differences, and additional support was provided to students needing extra time completing activities.

Consistent enforcement of webcam use presented a challenge. Initially, the teaching faculty could not agree on whether to require students to use a webcam during the live sessions. Thus, the use of a webcam was strongly encouraged, but not required, in Years One and Two. In Year Three, webcam use became mandatory after it was evident that several students were logged in with their cameras off, and not participating in any of the activities. This and other requirements were included in the agreement students signed at registration. That same year, we also experimented with a model of instruction in which students were assembled at one of the Tribal Colleges to increase student engagement. One of the Academy instructors, a respected faculty member, was on site to mentor students and addressed technical issues. Anecdotally, this cohort approach, though more complex logistically, led to fewer distractions and stronger group cohesion. This model may be adopted in future implementations of the course if additional faculty can be recruited to mentor students. Another Year Three change was the inclusion of more demonstrations and hands-on exercises in response to student feedback that instructional load was too high. While these additions were well-received, students continued to request more opportunities to exercise skills learned in the course.

Course participation requirements were minimal, with no restrictions on age, sex, academic interests or STEM experience. Given the heterogeneous backgrounds and motivations of students, an early challenge for faculty was identifying ways to sufficiently engage and differentiate instruction for students. Variability in performance on the student assessments in Year Three may partially explain student differences. Alternatively, the assessment questions may simply not have accurately matched the course content or may have reflected differences in how the faculty presented the material in the modules. Despite efforts to prepare students in advance for the Academy by ensuring that they had the required software and by guiding them through the process of gaining access to the learning management system, some had difficulty navigating the instructional technology. These difficulties were mitigated by the team’s instructional designer who often needed to liaise between the students and the university IT department.

Several recurring themes were evident all 3 years the course was offered and were consistent across assessment modalities. Course participants tended to view data science through an Indigenous lens. Students pointed to the potential benefit of data science to improve the lives of their families and communities. Students also enjoyed hearing from Indigenous health professionals who modeled the use of data to address real-world concerns of Indigenous people. While students requested more examples of Indigenous data use, they also expressed strong appreciation for the cultural relevance of the program.

Several innovative programmatic features distinguish the VIDS Academy from other STEM summer programs for underrepresented students. First, a participatory design approach ensured Tribal college faculty input at every stage of program development. For example, the decision to pursue a synchronous, online delivery format was in direct response to input from the Tribal college faculty. Additionally, the decision not to introduce a programming language to students was based on TCU faculty input. Second, the decision in Year Two to adopt a theme that was more congruous with the natural and environmental sciences expertise of faculty at the Colleges tapped into the experiences many students had working with College faculty. While this change represents a radical departure from more traditional biomedical applications of data science, it better resonated with students’ backgrounds. Studying the dynamics and ecology of mosquito-borne pathogens on population health avoided the need for IRB and Tribal research review--something that would have been unduly burdensome for a short summer program. It may be argued that, while students may have been seemingly more engaged with the course material, this shift in emphasis may also have been a factor in the decline in the number of participants from Year One to Year Three. The public health expertise of the UND SMHS faculty helped students understand the public health importance of climate change on mosquito-borne disease. Finally, the program embedded Indigenous perspectives by grounding the course in a cultural understanding of ethical data collection and use, in examples referencing Tribal data, and in the management of the course by both Indigenous and non-Indigenous faculty and peer mentors.

The VIDS Academy was created to meet the growing demand for data science and its application to public health challenges affecting Tribal communities in ND and beyond. The VIDS Academy offers a promising model for delivering data science and STEM instruction that aligns with local workforce and healthcare needs of Tribal communities. Future plans include transferring the VIDS Academy leadership to TCUs in ND and offering extended training in data science under a formal agreement with UND. An expanded data science curriculum is expected to create new avenues for the development of an innovative and impactful data science research agenda that is linked to issues of concern to the Indigenous population of the region and beyond.

## Data Availability

The raw data supporting the conclusions of this article will be made available by the authors, without undue reservation.
