# Supplementary material for: The virtual indigenous data science academy: development of a summer program in data science for tribal college students
Source: Front Public Health. 2026 Feb 12;14:1709106. doi: 10.3389/fpubh.2026.1709106 (PMC12936994; doi:10.3389/fpubh.2026.1709106)
Supplement: Supplementary file 1 [file Table_1.docx]

**Supplementary Material**

**Materials and Methods**

1. Scholar Responsibilities

VIDS Academy scholars must commit the time and effort required to complete distance-based activities (about five hours per week). Please sign if you agree to the following responsibilities:

- I will commit the time necessary to attend all Academy sessions.
- I agree to participate in group learning activities.
- I will read all assigned materials, complete assessments, and participate in distance learning activities.
- I have (or will have) timely access to computer hardware (not a tablet or mobile phone) and software with a webcam and reliable internet connection to participate in the distance-learning activities of the Academy.
- I understand that transportation to and from all Academy sessions at my Tribal College host site is my responsibility.

As a participant in the Virtual Indigenous Data Science Academy, I have read the description of “Scholar Responsibilities” and hereby commit and agree to all of the requirements of the Academy.

Signature: ________________________

2. Needs Assessment and Interest Survey

**Virtual Indigenous Data Science Academy**

**Needs Assessment and Interest Survey**

We are asking for your assistance so that we can better support Tribal college student participants in the 2022 summer *Virtual Indigenous Data Science Training Academy*.  The *Academy* is an NIH-funded partnership between participating North Dakota Tribal Colleges and the University of North Dakota School of Medicine and Health Sciences. Your input will help us design a training program that will fit best with the priorities of your Tribal college and the needs of your students. This survey consists of 21 questions and can be completed in approximately 10 minutes or less.

Corey B. Smith, PhD   
Co-director                 
 
Melanie Nadeau, PhD
Co-director
 
Please complete the following survey and return to us by **February 18, 2022**. Thank you.

Q1. Your name (optional)

________________________________________________________________

Q2. What is the name of your Tribal College?

- Cankdeska Cikana Community College
- Nueta Hidatsa Sahnish College
- Sitting Bull College
- Turtle Mountain Community College
- United Tribes Technical College

Q3. What is your age (in years)?

________________________________________________________________

Q4. Gender

- Man
- Woman
- Two Spirit
- Non-binary
- Gender Queer
- Another Gender Identity

Q5. What is your home department?

________________________________________________________________

Q6. Academic Rank (if applicable)

________________________________________________________________

Q7. How many years have you been working as a faculty member at the College?

________________________________________________________________

Q8. What courses do you teach, if any?

________________________________________________________________

Q9. Are there any faculty-led research projects in your department involving the collection and/or analysis of data that you would like us to know about for use by students in the data science academy?

________________________________________________________________

Q10. How effective do you think the following online course delivery formats are for instruction at your college?

|  | Not Effective (1) | Somewhat Effective (2) | Moderately Effective (3) | Very Effective (4) |
| --- | --- | --- | --- | --- |
| Online - asynchronous (1) |  |  |  |  |
| Online - synchronous (2) |  |  |  |  |
| Hybrid (3) |  |  |  |  |
| Blended synchronous (4) |  |  |  |  |

Q11. How many students from your Tribal College do you think are likely to enroll in a week-long summer training course on data science? Assume that students will receive a monetary incentive for completing each of 10 2-hour modules and a bonus for completing the entire course.

- 0 - 2
- 3 - 5
- 6 - 9
- 10 or more

Q12. How likely do you think the following may interfere with student participation in the data science training course?

|  | Very Unikely (1) | Unlikely (2) | Likely (3) | Very Likely (4) |
| --- | --- | --- | --- | --- |
| Job conflict |  |  |  |  |
| Family commitment |  |  |  |  |
| Transportation (car, gas money) |  |  |  |  |
| Other (please specify) |  |  |  |  |

Q13. How likely do you think the following may interfere with student completion in the data science training course?

|  | Very Unlikely (1) | Unlikely (2) | Likely (3) | Very Likely (4) |
| --- | --- | --- | --- | --- |
| Job conflict |  |  |  |  |
| Family commitment |  |  |  |  |
| Transportation (car, gas money) |  |  |  |  |
| Other (please specify) |  |  |  |  |

Q14. How effective do you think the following incentives would be for recruiting students to the data science training course?

|  | Not Effective (1) | Somewhat Effective (2) | Moderately Effective (3) | Very Effective (4) |
| --- | --- | --- | --- | --- |
| UND course credit |  |  |  |  |
| Certificate of completion |  |  |  |  |
| Payment for completion |  |  |  |  |
| Other (please specify) |  |  |  |  |

Q15 The following 4 questions ask about computing resources at your Tribal college.  At your Tribal college...

a. Do all students have access to a computer or workstation that is less than 3 years old?

- Yes
- No
- Don't know

b. Is it necessary that students be on campus to have access to broadband service in your community?

- Yes
- No
- Don't know

c. Which of the following data management and statistical software applications are students familiar with? (Please check all that apply.)

- ArcGIS
- Excel
- MS Access
- SPSS
- SAS on demand for academics
- R
- Python
- None of the above
- Other (please specify) __________________________________________________

d. Which learning management systems do you use for the administration and delivery of courses? (Please check all that apply.)

- Blackboard
- Canvas
- Desire2Learn
- Moodle
- Other (please specify) __________________________________________________

Q16. We will be using real-world examples to illustrate key concepts in the data science training course. Which of the following subject areas do you think would have the greatest appeal to students wanting to better understand data science? (Please check all that apply.)

- Sports
- Education
- Entertainment
- Finance
- Omics (genomics, proteomics, phenomics, etc.)
- Health or healthcare
- Environment
- Tourism
- Other (please specify) __________________________________________________

Q17. What questions do you have about our program that you would like us to answer?

________________________________________________________________

3. Learning Objectives and Modules Matrix

| Learning Objective | Module | | | | | | | | | |
| --- | --- | --- | --- | --- | --- | --- | --- | --- | --- | --- |
|  | 1 | 2 | 3 | 4 | 5 | 6 | 7 | 8 | 9 | 10 |
| 1. Describe the interdisciplinary field of data science | ✓ |  |  |  |  |  |  |  |  |  |
| 2. Discuss Indigenous perspectives of health data ethics and implications for TCUs and Tribal communities |  | ✓ |  |  |  |  |  |  |  |  |
| 3. Explain the relationship of climate, mosquito ecology, and disease prevalence (e.g., West Nile Virus) |  |  | ✓ |  |  |  |  |  |  | ✓ |
| 4. Review foundational concepts in probability and statistics for data analytics |  |  |  |  | ✓ |  |  |  |  |  |
| 5. Describe the role of statistics in data science |  |  |  |  | ✓ | ✓ |  |  |  |  |
| 6. Differentiate between spreadsheets and database systems |  |  |  | ✓ |  |  |  | ✓ |  |  |
| 7. Match a programming language to a data science application |  |  |  |  |  | ✓ |  |  |  |  |
| 8. Apply guidelines for scientific visualization using MS Excel to summarize different types of data |  |  |  |  |  |  | ✓ |  |  | ✓ |
| 9. Gain programming experience using MS Excel to manage, explore, and visualize data |  |  | ✓ |  |  |  |  |  |  | ✓ |
| 10. Describe types of studies for investigating research questions |  |  |  |  |  |  |  | ✓ | ✓ |  |
| 11. Distinguish between Big Data and little data |  |  |  |  |  |  |  | ✓ |  |  |
| 12. Describe the role of analytics in the transformation of data to knowledge |  |  |  |  |  |  |  | ✓ |  |  |
| 13. Weigh the advantages and disadvantages of cloud computing in biomedical research | ✓ |  |  |  |  |  |  | ✓ |  |  |
| 14. Outline the steps of an analytic process |  |  |  |  |  |  |  | ✓ | ✓ |  |
| 15. Recognize common applications of machine learning in healthcare |  |  |  |  |  |  |  |  | ✓ |  |
| 16. Interpret results of a machine learning solution |  |  |  |  |  |  |  |  | ✓ |  |
| 17. Demonstrate understanding of the potential and limitations of algorithmic knowledge |  | ✓ |  |  |  |  |  |  | ✓ |  |

4. Talking Circle Protocol

1. Respect the talking piece so the person who has it can convey their full message without interruption. (A talking piece is s a meaningful and symbolic object that the facilitator, also called the “circle keeper,” brings to the circle. A talking piece was not used in this online format. Instead, the circle keeper regulated the conversation.)

2. When you don’t have the talking piece, listen respectfully and reflect upon, consider, and honor the meaning of what others say so you can build on the conversation.

3. You can pass if you need to. Nonverbal communication and silence sometimes say more than words.

4. Speak for yourself and from your own experiences and perspectives. Use “I language” and not generic “people think. . .” or sweeping generalizations, like “students want. . .” language.

5. Be courageous, honest, and open with your own stories. Speak your truth from your heart and be open to hearing others’ truths.

6. Listen from the heart, allowing what others say to move you. Bear witness but do not provide advice or argue with others.

7. Honor what others say with confidentiality and integrity, sharing only with context and in relevance to your own life and learning, not as gossip.

5. Summative Assessment Questions

**1. Foundations of Data Science**

Q1 Which of the following is not one of the stages in the Data Science Life Cycle?

- Plan
- Analyze
- Implement
- Communicate

Q2 Data scientists are concerned with all of the following except:

- Wisdom
- Data
- Information
- Knowledge

Q3 Data science uses AI to teach computers how to mimic human behavior.

- True
- False

Q4 Compliance with the FAIR principles ensures that data can be easily shared.

- True
- False

**2. Cultural Aspects of Data Collection and Use***

*****In lieu of polling questions, this module consisted of a group exercise involving the review of an article.

**3. Mosquitoes, Climate, and Health**

Q1 Which of the following mosquito species are the main vectors for West Nile Virus (WNV)?

- Aedes vexans
- Culex tarsalis
- Aedes dorsalis
- Anopheles genera

Q2 Which of the following is true of zoonotic transmission of disease?

- An animal infected with a zoonotic pathogen is free from symptoms
- Zoonotic transmission can only occur through insect bites
- Zoonotic diseases are transmitted from wildlife to humans
- All of the above

Q3 Which of the following is not true of West Nile Virus (WNV)?

- WNV is a vector-borne disease
- WNV is capable of zoonotic transmission
- WNV can be transmitted between humans
- WNV cycles between mosquitoes and birds

Q4 Which of the following traps is designed to attract and catch West Nile Virus spreading species?

- CO2 traps
- Resting boxes
- Ovide traps
- Gravid traps

**4.** **Overview of Data Sources**

Q1 Which of the following is not a NEON data product?

- Mosquito samples
- DNA sequences
- Reservoir hosts
- Pathogen status

Q2 Climate affects mosquito populations in the following ways except:

- Mode of transmission
- Habitable territory
- Breeding opportunities
- Transmission season

Q3 A case is defined as a person who tests positive for disease as determined by a licensed physician

- True
- False

Q4 West Nile Virus is a reportable disease according to the state of North Dakota but not the CDC

- True
- False

5. Understanding Distributions, Statistics, and Hypothesis Testing

Q1 What is the difference between continuous and categorical variables?

________________________________________________________________

Q2 What is the difference between a population and a sample?

________________________________________________________________

Q3 Why do we need to calculate confidence intervals?

________________________________________________________________

Q4 Which of the following is true about a hypothesis?

- A hypothesis is a statement that should never be rejected
- A hypothesis is a testable statement that can never be rejected
- A hypothesis is a testable statement that can be accepted or rejected based on the results of statistical testing
- None of the above are true

**6.** **Data Management and Processing**

Q1 What is SAS/SODA?

________________________________________________________________

Q2 Describe the structure of a dataset

________________________________________________________________

Q3 We talked today a lot about statistical procedures. Describe in your own words the purpose of a statistical procedure.

________________________________________________________________

Q4 Proc print and proc contents are statistical procedures in SAS.

- True
- False

**7.** **Programming and Tools for Data Science**

Q1 High level programming languages must be translated into machine language for the computer to understand.

- True
- False

Q2 Which of the following are disadvantages to using spreadsheets for data science?

- Slower processing speed
- Fewer analytic capabilities
- Limited graphics and visualization options
- All of the above

Q3 Which of the following is not an example of an open-source programming language?

- Python
- Perl
- SAS
- R

Q4 All of the following are advantages of Microsoft Excel except:

- Ability to document steps in analysis
- Easy to get started
- Has free add-on packages
- Works with other Microsoft applications

**8. Analytics and Big Data in Healthcare**

Q1 The five Vs are used to tell the difference between Big data and small data.

- True
- False

Q2 Predictive analytics are used to forecast what will happen in the future.

- True
- False

Q3 Which of the following is the most significant technical challenge in any big data ecosystem?

- Integration
- Management
- Analytics
- All of the above

Q4 Which of the following is considered a disadvantage of cloud computing?

- Availability
- Privacy
- Dependence on the internet
- Low maintenance

**9. Introduction to Machine Learning**

Q1 Unsupervised learning requires a training dataset in order to learn to identify patterns in data.

- True
- False

Q2 Machine learning solutions are vulnerable to all of the following biases except:

- Training dataset
- Interpretability
- Coding of algorithms
- Application of results

Q3 Genome Wide Association Studies begin with data on phenotype.

- True
- False

Q4 Which of the following is not true of algorithms?

- Algorithms are subject to bias
- Algorithms are easily interpreted
- Algorithms are sensitive to noisy data
- Algorithms drive the modeling process

**10.** **Communicating with Data**

Q1 How many Native Elders who were 55 years or older participated in the, “Identifying Our Needs: A Survey of Elders” cycle 8 assessment from the data booklet?

- 23,602
- 10,505
- 19,744
- 3,560

Q2 Which three states had the highest number of Indigenous communities participate in the cycle 8 needs assessment?

- North Dakota, Florida, and Texas
- Alaska, California, and Oklahoma
- Oregon, South Dakota, and Washington
- Maine, New York, and Arizona

Q3 What three states had the highest percentage of Native Elders participate in the cycle 8 needs assessment survey?

- Massachusetts, New Mexico, and North Carolina
- Wisconsin, Nevada, and Kansas
- Louisiana, Illinois, and Virginia
- Oklahoma, Arizona, and New Mexico

Q4 What were the top five most frequently reported health conditions from the cycle 8 survey?

- Congestive Heart Failure, Asthma, Osteoporosis, Diabetes, and Cataracts
- Stroke, Depression, Arthritis
- High Blood Pressure, Arthritis, Diabetes, Cataracts, and Depression
- Cataracts, Prostate Cancer, Congestive Heart Failure, and Diabetes
