# Supplementary material for: The virtual indigenous data science academy: development of a summer program in data science for tribal college students
Source: Front Public Health. 2026 Feb 12;14:1709106. doi: 10.3389/fpubh.2026.1709106 (PMC12936994; doi:10.3389/fpubh.2026.1709106)
Supplement: Supplementary file 2 [file Table_2.docx]

**Supplementary Material**

**Materials and Methods**

1. Scholar Responsibilities

VIDS Academy scholars must commit the time and effort required to complete distance-based activities (about five hours per week). Please sign if you agree to the following responsibilities:

- I will commit the time necessary to attend all Academy sessions.
- I agree to participate in group learning activities.
- I will read all assigned materials, complete assessments, and participate in distance learning activities.
- I have (or will have) timely access to computer hardware (not a tablet or mobile phone) and software with a webcam and reliable internet connection to participate in the distance-learning activities of the Academy.
- I understand that transportation to and from all Academy sessions at my Tribal College host site is my responsibility.

As a participant in the Virtual Indigenous Data Science Academy, I have read the description of “Scholar Responsibilities” and hereby commit and agree to all of the requirements of the Academy.

Signature: ________________________

Major areas of study for VIDS participants, 2022-2024 (N = 48).

|  | **VIDS 1.0**  **(N=19)** | | **VIDS 2.0**  **(N=16)** | | **VIDS 3.0**  **(N = 13)** | | **Total**  **(N = 48)** | |
| --- | --- | --- | --- | --- | --- | --- | --- | --- |
|  | N | %^a^ | n | %^a^ | n | %^a^ | n | %^a^ |
| Natural Sciences (Biology, Chemistry, Other Science) | 2 | 10.5 | 3 | 18.7 | 4 | 30.7 | 9 | 18.7 |
| Environmental Sciences | 5 | 26.3 | 0 | 0.0 | 2 | 15.4 | 7 | 14.5 |
| Humanities (Fine Arts, Liberal Arts) | 3 | 15.8 | 2 | 12.5 | 1 | 7.7 | 6 | 12.5 |
| Undeclared | 1 | 5.3 | 2 | 12.5 | 2 | 15.4 | 5 | 10.4 |
| Education | 2 | 10.5 | 1 | 6.3 | 1 | 7.7 | 4 | 8.3 |
| Behavioral and Social Sciences (Counseling, Social Work, Psychology) | 1 | 5.3 | 1 | 6.3 | 1 | 7.7 | 3 | 6.3 |
| Business (Accounting, Business Administration, Business Management) | 0 | 0.0 | 3 | 18.7 | 0 | 0.0 | 3 | 6.3 |
| Indigenous Studies and Food Systems | 1 | 5.3 | 1 | 6.3 | 1 | 7.7 | 3 | 6.3 |
| Computer Science/Information Technology | 1 | 5.3 | 1 | 6.3 | 0 | 0.0 | 2 | 4.2 |
| Health Professions (Medical Lab Technology, Public Health) | 2 | 10.5 | 0 | 0.0 | 0 | 0.0 | 2 | 4.2 |
| Pre-engineering | 0 | 0.0 | 2 | 12.5 | 0 | 0.0 | 2 | 4.2 |
| Nursing | 1 | 5.3 | 0 | 0.0 | 1 | 7.7 | 2 | 4.2 |
| Mathematics | 0 | 0.0 | 0 | 0.0 | 0 | 0.0 | 0 | 0.0 |

Percent of correct responses on VIDS 3.0 student learning assessments (N = 13)

| Module | | | | | | | | | |
| --- | --- | --- | --- | --- | --- | --- | --- | --- | --- |
| Question | **1**  **(n=12)** | **3 (n=13)** | **4**  **(n=12)** | **5**  **(n=10)** | **6**  **(n=9)** | **7**  **(n=11)** | **8**  **(n=13)** | **9**  **(n=11)** | **10**  **n=11)** |
| 1 | 92% | 62% | 33% | 60% | 56% | 91% | 79% | 36% | 91% |
| 2 | 50% | 54% | 67% | 70% | 75% | 82% | 7% | 27% | 100% |
| 3 | 58% | 100% | 0% | 70% | 78% | 36% | 79% | 73% | 100% |
| 4 | 92% | 23% | 67% | 100% | 89% | 64% | 57% | 55% | 82% |
| Overall correct responses | **73%** | **60%** | **42%** | **75%** | **75%** | **68%** | **56%** | **48%** | **93%** |
